# Outcomes of the NCI cancer prevention fellowship program: a focus on mentorship within a multidisciplinary public health field

**DOI:** 10.1186/s12909-026-09387-1

**Published:** 2026-05-13

**Authors:** Shanen M. Sherrer, Jessica M. Faupel-Badger, Krista A. Zanetti, Heather R. Bowles, Philip E. Castle

**Affiliations:** 1 Cancer Prevention Fellowship Program, National Cancer Institute, Bethesda, MD USA; 2 Present Address: Department of Chemistry and Biochemistry, AAAS Science & Technology Policy Fellow, St. Mary’s College of Maryland, St. Mary’s City, MD USA; 3 Division of Research Capacity Building, National Institute of General Medical Sciences, Bethesda, MD USA; 4 Office of the Director, National Institutes of Health, Bethesda, MD USA

**Keywords:** Public health postdoctoral training, Mentorship, Evaluation, Cancer Prevention, Multidisciplinary training

## Abstract

**Background:**

Mentorship, a component inherent in postdoctoral training programs, is vital for professional development and retention of early-career researchers. Yet, it remains difficult to measure the impact of mentorship on one’s career. In this study, the National Cancer Institute’s (NCI) Cancer Prevention Fellowship Program (CPFP) alumni spanning from 1987 to 2024 were surveyed about their career outcomes, and the impacts of their mentorship experience also emerged.

**Methods:**

A survey was sent by email to CPFP alumni through Qualtrics with reminders during the period of May 14 through July 12, 2024. Of the 261 alumni recruited to complete the survey, 189 (72%) responded.

**Results:**

When reflecting on their multidisciplinary training in cancer prevention and control research, they valued both mentoring and networking aspects of the program (82.7%), and 46.0% and 68.8% of alumni said that CPFP was at least very beneficial to their mentoring skills and professional networking skills, respectively. At the time of this study, alumni were likely to complete all postdoctoral training (99.5%), with 74.7% completing their training within 2 to 5 years. Most alumni also worked across all public health job sectors (87.8%), while being mentors who spent time advising students and fellows (84.9%), led or contributed to multidisciplinary training programs (71.9%), and/or served as mentor to others in their organization (96.6%).

**Conclusions:**

The survey data demonstrate CPFP alumni valued their mentoring experiences in this postdoctoral program and now serve in mentoring roles themselves. CPFP alumni are also being retained in the field of cancer prevention and control and continue to build the workforce.

**Clinical trial number:**

Not applicable.

**Supplementary Information:**

The online version contains supplementary material available at 10.1186/s12909-026-09387-1.

## Background

Mentorship is an important component of professional development, especially for public health training [1]. However, the definition of mentorship varies based on needs, resources, and policies. Mentorship can be described as a simple practice in which a person with experience (“mentor”) provides support and advice to a less experienced person (“mentee”) in the profession [2]. Conversely, mentorship is often confused with the concept of sponsorship for a mentee. A mentor is normally someone who gives advice with possible transactional expectations while sponsorship provides valuable opportunities to the mentee, using the mentor’s network to advocate for the mentee, without additional commitments [3].

When supporting scientific mentorship at any career stage, there are several points to consider. For example, both the mentor and mentee within the relationship will have increased access to professional development and broaden participation in research areas such as public health [3–6]. In addition, increased retention of the workforce can be achieved by having a positive impact on a mentee’s self-identity and increasing the mentee’s personal motivation to continue in public health-related fields [2, 3, 7]. These competencies have been linked to the practice of encouraging early-career scientists to be very active within scientific and professional societies [8], which in turn allow for timely career advancements. To achieve highly effective mentorship, mentors may need to be comfortable with being in uncomfortable scenarios [9], and it may come at a personal cost [e.g., little to no service recognition towards mentorship, redistribution of time and/or efforts for other commitments, and other professional outcomes considered not a career punishment] to mentors [3]. However, by supporting meaningful mentorships, mentors can influence the direction of the scientific field and strengthen the personnel infrastructure to advance research [3, 8].

Postdoctoral scholars are defined herein as someone doing scholarly and/or research work and/or training after earning a PhD- and/or MD-equivalent degree prior to starting an entry-level career position. Notably, the definition of postdoctoral scholars from the National Postdoctoral Association also includes the detail that they are in a temporary period of mentored research to acquire needed professional skills for entry-level career jobs [10]. Snapshots of current trends in mentorship often include relationships that are ambiguous and informal for postdoctoral scholars in science, technology, engineering, mathematics, and medicine (STEMM) fields such as public health [3, 7]. These observations of mentoring trends are possibly based on outdated career predictions and opaque outcome communications for postdoctoral scholars [11]. For mentors, the effort put into these relationships may not be valued at the institutional level. It is assumed research mentors will provide scientific and technical skills training but other career development competencies such as research program management and high-impact communication skills may be less emphasized and equally important [8]. Mentors also may not be fully aware of professional development opportunities that would complement and benefit the postdoctoral mentee’s research and career endeavors [4, 8, 12]. In an ideal setting, postdoctoral mentorship should involve a two-way exchange of sharing and learning [3, 9].

The aforementioned mentorship trends provide justification for postdoctoral programs to promote skill development beyond scientific and technical training. For example, early-career participants value programs that include components such as protected time, collaborations, networking, rigorous grant writing activities, and different types of formal mentoring [13]. Here, the mentoring opportunities and impacts of the National Cancer Institute’s (NCI) Cancer Prevention Fellowship Program (CPFP) were examined. CPFP is an on-site postdoctoral training program at NCI that was established in 1987 to recruit multidisciplinary talent and to train future leaders in the public health field of cancer prevention and control [14, 15]. To date, CPFP survey studies have been conducted in 2014 and 2024. While the main goal of these surveys was to measure career outcomes of program alumni [14, 15], mentorship experiences and impacts within CPFP became visible in alumni responses during data analysis of the 2024 survey. These 2024 data support the value of effective mentorship at the postdoctoral level within a training program as well as progression towards addressing a complex public health issue such as preventing cancer.

## Methods

### Study populations

The same methods as described in Sherrer et al. [15] were performed for this study. In brief, the study population included CPFP alumni (*n* = 355) and applicants (*n* = 569) from 1987 to 2024. Since 1991, eligible CPFP alumni participants were given the opportunity to earn an MPH degree sponsored by NCI during the first year of their program and received up to four years of mentored multidisciplinary postdoctoral research support and training. Importantly, the CPFP cohort that started in 2019 completed their program training by 2024. Please note that the control group named ‘CPFP applicants’ were those who were invited to the interview step of the CPFP application but did not participate in the program. While some of the CPFP applicants chose not to participate in the program, the majority of the applicants were not offered the fellowship. The entire CPFP application pool of this time frame was not included to best reflect and compare individuals who valued aspects of postdoctoral training programs like CPFP, had competitive CPFP applications, and wanted a career in the field of cancer prevention and control.

### Instrument development and survey implementation

The 2024 survey instrument, which was the same as the 2011 CPFP survey instrument [14] with slight modifications, was designated institutional review board (IRB) exempt (#2024-066) and was administered via Qualtrics online. The 2024 survey can be viewed in the accompanying *Scientific Reports* article [15], which included updated demographic questions as well as new questions on postdoctoral timeline, work achievements, current job titles, and affiliations with NCI-designated cancer centers. All survey questions were optional and included topics on demographics, employment, career activities, professional association and awards, program benefits, and program reflection and recommendation inquiries. The length of the survey instrument was controlled to reduce chances of response fatigue, which in turn limited scope of questions addressing any aspects of mentorship. A third-party service company (ICF) was contracted to obtain the IRB approval, administer this survey, solicit participation in the survey from eligible CPFP cohorts and applicants, collect survey responses which included informed consent from participants, and aggregate and code survey data for the authors of this study.

### Outcome measurements and statistical analysis

Calculations were based on tabulations on aggregated themes. When appropriate, bivariate analysis using Fisher’s exact tests were performed to calculate distribution differences between alumni responses and applicant responses for selected variables. To ensure assessments of mentorship impacts were as comprehensive as possible, collaboration and research networking activities were included in the analysis when possible.

## Results

### Survey respondent demographics

The study included two survey participation populations – respondents who completed CPFP prior to the administration of the survey in 2024 (hereafter called ‘alumni’) and respondents who were qualified and interviewed for admission into CPFP but were not enrolled into the program mostly because they were not offered one of the limited number of positions per cohort (hereafter called ‘applicants’). One hundred and eighty-nine (72.4%) CPFP alumni responded to the 2024 survey request when compared to 52 (16.2%) applicants. Within the respondent populations, there are no statistical differences in race/ethnicity and sex between alumni and applicants (Table 1 and Sherrer et al. [15]). The distribution of postdoctoral completion cohorts (all position types) were not identical for alumni and applicants albeit insignificant statistically in differences when comparing year to year of completion (Supplementary Fig. S1 and Sherrer et al. [15]).

**Table 1:** Self-selection of race/ethnicity of survey respondents^a^

Race/Ethnicity	Percent of CPFP alumni (*n* = 189)	Percent of CPFP applicants (*n* = 52)
White	72.1	63.8
Asian	11.0	*
Black, African American, or African	8.7	*
Other^b^*	15.7	*

^a^Data derived from Sherrer et al. [15]

^b^’Other’ included Hispanic, Latino, or Spanish; Middle Eastern or North African; American Indian or Alaska Native; Native Hawaiian or Other Pacific Islander; none of the above option; and prefer not to answer option

*Due to *n* < 10, data was suppressed or collapsed into a larger category to reduce risk of identity of survey respondents

### Length of transitions into careers

In terms of length of transition time and corresponding career outcomes, there were subtle differences between CPFP alumni and applicants. For example, alumni stayed in postdoctoral positions longer (Table 2, 2–5 years including completion of CPFP) than applicants (1–4 years) (*p* = 0.01). Note that these data did not differentiate between respondents who did not complete a postdoctoral position due to career changes from those who entered directly into an entry-level employment after completion of their highest degree. Within their postdoctoral positions, mentorship training and mentoring experiences with supervisors and/or mentors were reported for both CPFP alumni and applicants, and the corresponding data were further analyzed below.

**Table 2:** The postdoctoral training amount and length of CPFP alumni and applicants

Category	Percent of CPFP alumni	Percent of CPFP applicants	Fisher’s exact test
Number of completed training programs/positions			
Not completed a postdoctoral or fellowship program/position	*	25.0	
One postdoctoral or fellowship program/position	78.0	59.6	
Multiple postdoctoral or fellowship program/position	21.2	15.4	
* Total n*	*189*	*52*	*p* < 0.01
Years in postdoctoral training			
No years	*	*	
1–2 years	*	12.8	
2–3 years	16.2	28.2	
3–4 years	31.7	28.2	
4–5 years	26.8	*	
5–6 years	11.3	*	
6–10 years	11.2	12.9	
* Total n*	*186*	*39*	*p* = 0.01
Work within scientific disciplines^a^			
1 discipline	48	57	
2 disciplines	34	39	
3–6 disciplines	19	*	
* Total n*	*181*	*49*	*p* = 0.26

^a^Data derived from Sherrer et al. [15]

*Due to *n* ≤ 10, data was suppressed to reduce risk of identity of survey respondents

### Immediate mentorship impact of postdoctoral positions

**Fig. 1:**
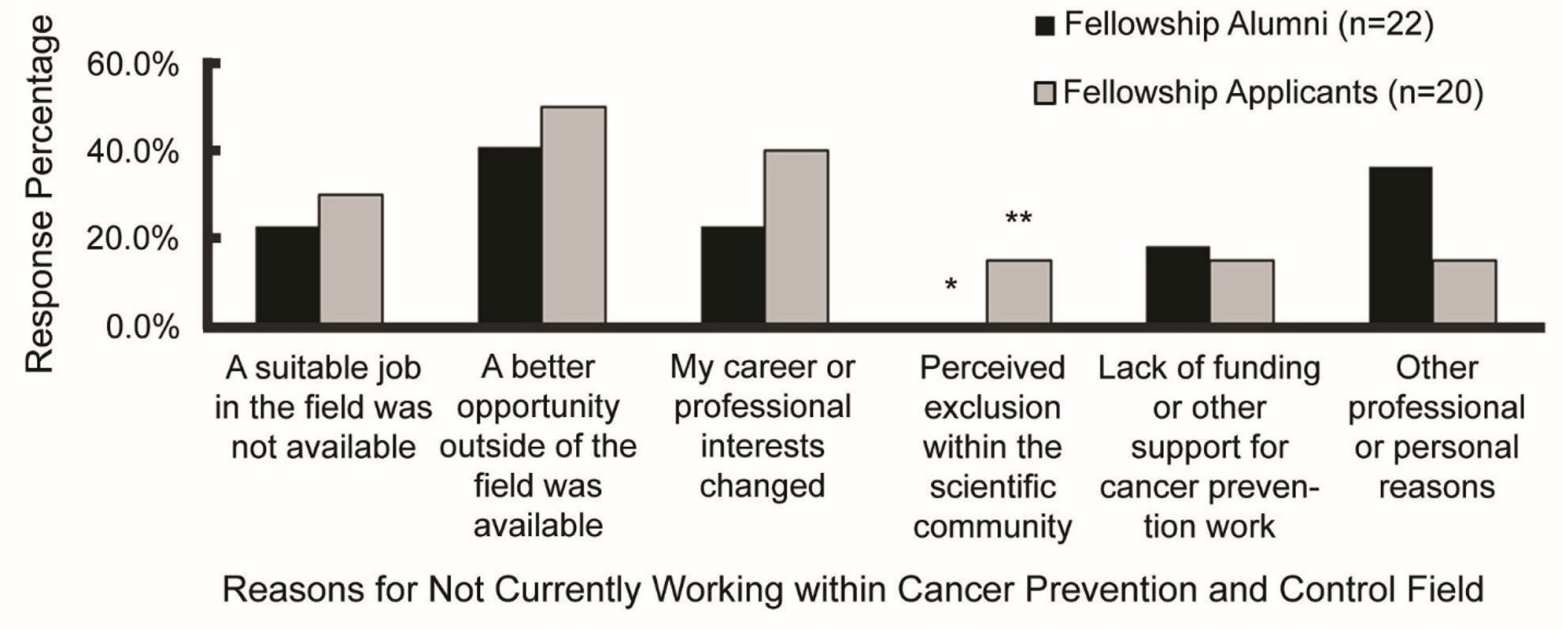
The majority of respondents who do not work within cancer prevention and control cited that they found better opportunities available outside of the field. While sample sizes are small compared to overall number of responses, the ratios on exact reason(s) for not working within cancer prevention and control (x-axis) differed between CFPF alumni (*n* = 22, black bars) from applicants (*n* = 20, grey bars). The * mark denotes data suppression due to small response amounts (*n* < 10). The ** mark denotes statistical differences in response between CPFP alumni and applicants on specific reason for not being in cancer prevention and control field with *p* = 0.1 for a two-sided Fisher’s exact test

To elucidate the impact of mentored research experiences, work activities and career outcomes were examined. At the end of their postdoctoral training, CPFP alumni were more likely to work within more than one scientific discipline than applicants (Table 2). As previously reported [15], the majority of these alumni (*n* = 121, 67.0%) worked within epidemiology and/or public health while the applicants (*n* = 21, 43.0%) worked in behavioral or social science. However, there were a very small number of CPFP alumni (*n* = 22, 11.6% of alumni respondents) and noticeable number of applicants (*n* = 20, 38.5% of applicant respondents) who were not currently working within cancer prevention and control (Fig.1). Within this population of respondents, 15.0% of CPFP applicants and 0.0% of alumni (*p* = 0.1) reported not currently working in the field of cancer prevention and control due to perceived exclusion within the scientific community. The most common reason given by both CPFP alumni (40.9% of *n* = 22) and applicants (50.0% of *n* = 20) for not working in the field was that there were better opportunities outside of the field. Due to low response rate of applicants for their open-ended question on accomplishments (*n* = 15, 28.8% of applicant respondents), the remainder of this study focused on mentoring aspects of CPFP alumni.

### Mentorship and networking experiences within CPFP

To understand the mentorship experiences and training during CPFP, the alumni were asked an open-ended question about the most valuable aspect of the CPFP to their career in the *Reflections and Recommendations* section of the survey [15]. Alumni frequently indicated (Table 3) that they valued mentoring and networking aspects of the program (82.7%), including receiving *exceptional mentorship* from NCI colleagues and CPFP leadership (35.5%). As examples, alumni shared their experiences on valuable mentoring aspects of CPFP (see below):

- *“[…] to have that training reinforced by amazing mentorship at the NCI was such a special combination.”*
- *“The mentoring I received was incredible – it inspired my work*,* my job*,* my research trajectory*,* my own mentoring.”*
- *“Connecting with [person’s] mentor.”*

**Table 3:** Valued aspects of program reported by CPFP alumni^a^

Valuable Aspect Themes	Theme Summaries
Mentorship and Networking (*n* = 62)	• Exceptional mentorship from NCI colleagues and CPFP leadership was frequently mentioned as a cornerstone of the fellowship experience (*n* = 22). • Building connections with peers, senior scientists, and professionals across various disciplines was invaluable for career advancement and collaboration (*n* = 44).
Personal Development and Opportunities (*n* = 13)^b^	• The fellowship provided the time and support needed to transition to new fields or research areas, helping fellows to redefine their career paths (*n* = 3). • The experience boosted fellows’ confidence and provided opportunities for independent learning and exploration (*n* = 10).

^a^Because this was an open response option, the responses were coded into overarching themes presented in the table above

^b^Data derived from Sherrer et al. [15]

CPFP alumni also considered highly valuable (71.0%) the networking potential offered through the program that included peers, senior scientists, and professionals across multiple disciplines when pursuing cancer prevention and control research projects. For example, alumni cited the single most valued aspect of CPFP to be:

- *“The mentorship and the relationship with the fellows.”*
- *“The support of the other fellows and at the time*,* having mentors who [I] did not direct[ly] work on projects with. There were no conflicts of interests with their advice. I felt their advice was truly [beneficial to] me.”*
- *“Networking with my cohort and other cohorts of CPFP fellows and mentors.”*

Alumni reported aspects of CPFP that were extremely or very beneficial (Supplementary Fig. S2) to their mentoring skills (46.0%), leadership and/or management skills (52.6%), professional networking skills (68.8%), and contacts who advised or collaborated with them on their research (72.9%). The mentorship experiences that occurred through professional networks during their time in CPFP led to personal growth and were also highlighted in the open-ended responses. Below is one example:

*“One extremely valuable aspect [of CPFP] was exposure – exposure to new ideas*,* to big picture thinking*,* to content experts*,* to potential collaborators and vibrant professional network*,* to the ins and outs of the extramural funding process*,* and to excellent mentoring. Those exposures were incredibly important in giving me the skills*,* and the confidence*,* to pursue my career post fellowship.”*

This professional confidence based on mentorship skills and potential networking was evident across CPFP cohorts, which was illustrated by a respondent’s program improvement suggestion shown below:

*“One possibility is to invite alumni to serve in some capacity as mentors/advisers for new fellows based on research interest and career goals; would bring a perspective and insights that might not be present through the program or NCI mentors.”*

Approximately 77% of alumni reported they highly valued the personal growth (Table 3) experienced during CPFP that boosted self-confidence and provided opportunities for exploration. This personal growth experienced by CPFP alumni was consistent with the low frequency of responses for not being in the cancer prevention and control field due to identity in the community, lack of funding or other support for cancer research, and/or professional interest changes (Fig. 1).

### CPFP alumni on the job as mentors and advisers

**Fig. 2:**
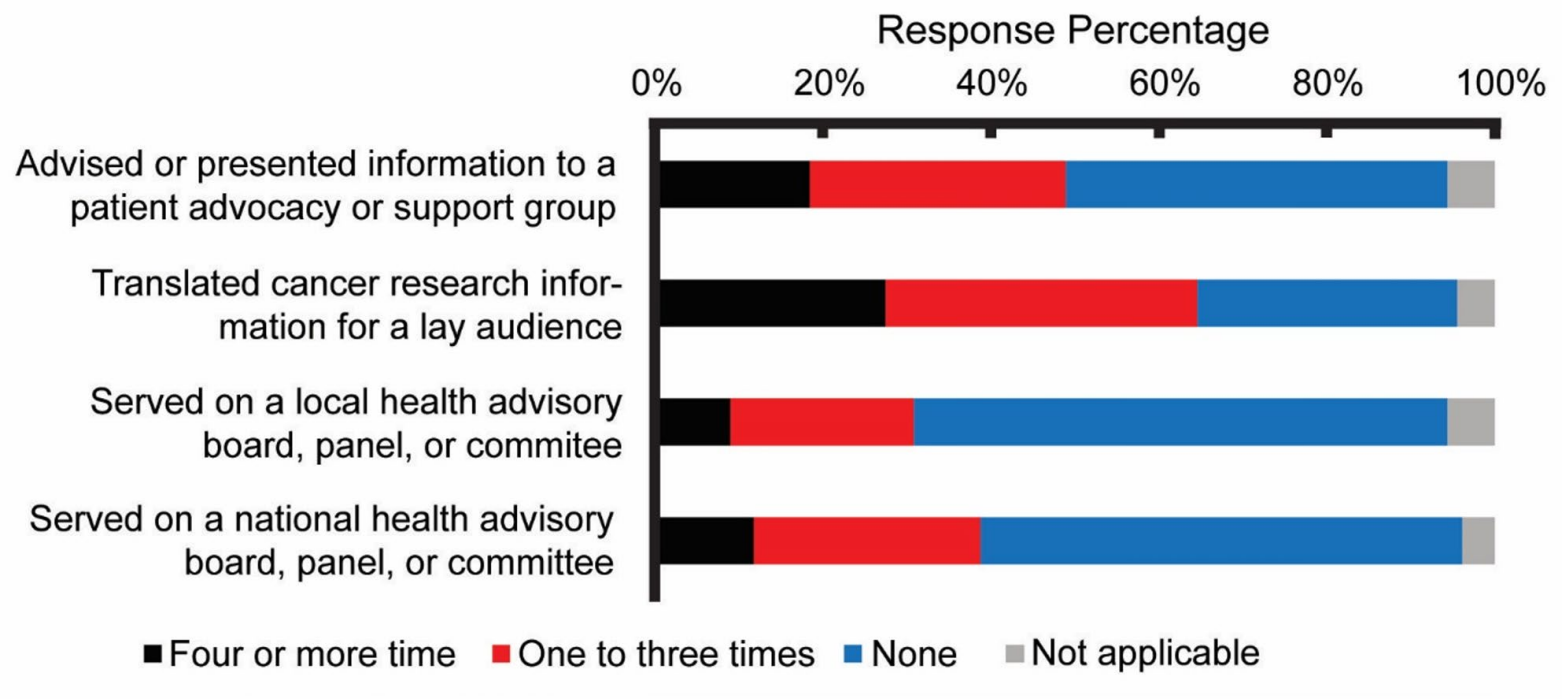
CPFP alumni perform services that are relevant to public health. Within the alumni’s current positions, related community service activities were rated in frequency of specific tasks (vertical items) as small or moderate extent (red bars); a very large or large extent (black bars); not at all (blue bars); or not applicable (grey bars)

**Fig. 3:**
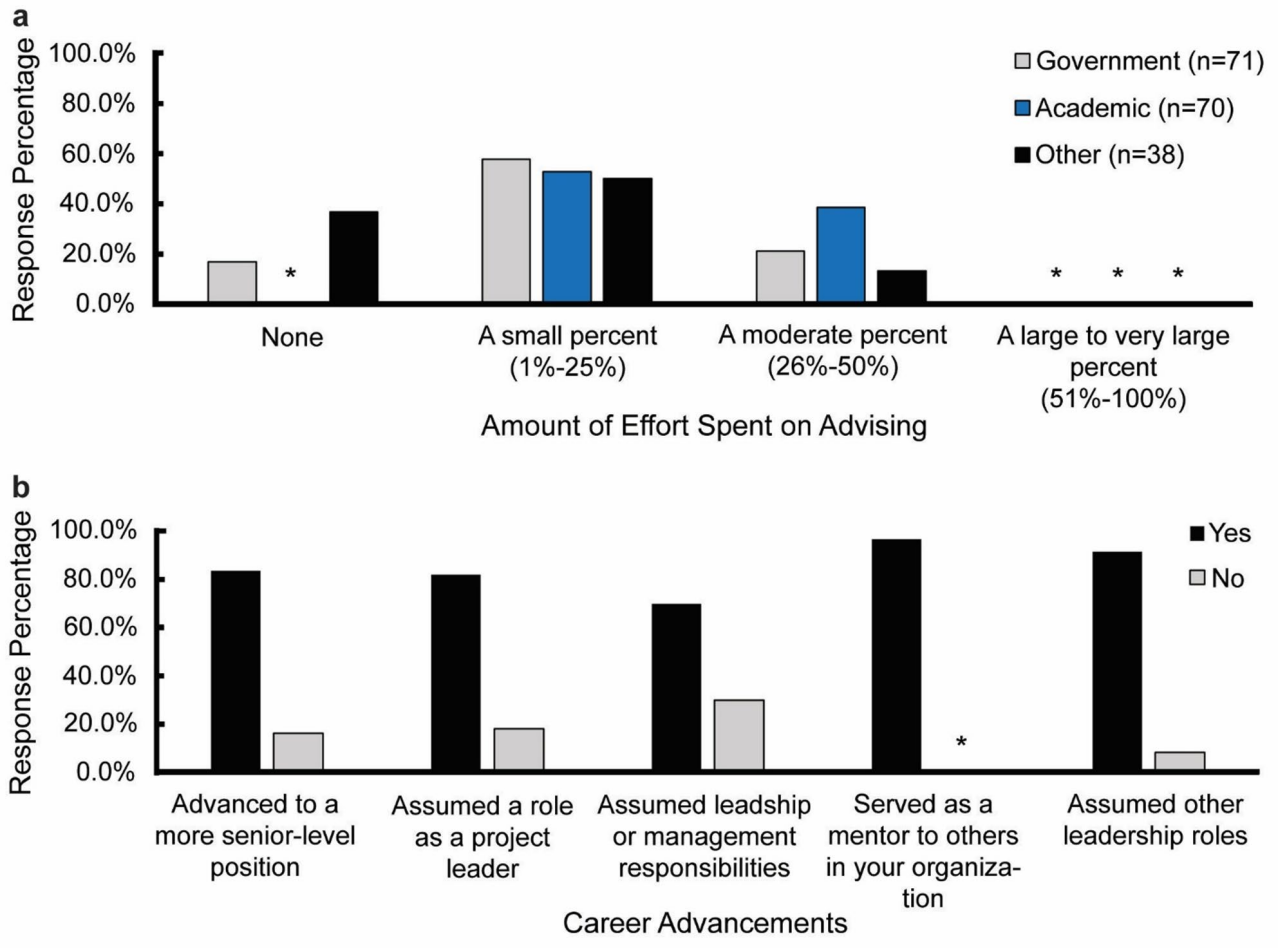
CPFP alumni puts effort into leading, mentoring, and advising staff, students, and fellows. (a) Alumni advise students and fellows within the government sector (*n* = 71, grey bars), academia (*n* = 70, blue bars), and other job sectors (*n* = 38, black bars). The portion of effort that CPFP alumni spent advising students and fellows were grouped by advising time for current employment – none (0% of time), a small percentage (1-25% of time), moderate percentage (26-50% of time), large percentage (51-75% of time), and very large percentage (76-100% of time). (b) Since completing their doctoral degree and postdoctoral work, CPFP alumni reported “yes” (black bars) or “no” (grey bars) to the career advancements indicated within the graph. For both graphs a and b, the * mark denotes data suppression due to small response amounts (*n* < 10)

Of the 87.8% of alumni from the 2024 CPFP survey who were retained in the field of cancer prevention and control, these individuals were in all job sectors (39.5% academia, 39.4% government, and 10.3% other). Within the past five years of their careers (Fig. 2), alumni performed public health-related activities at least once that require mentoring and advising skills. Examples of these activities include translating cancer research information for a lay audience (64.6%) and serving on a national health advisory board, panel, or committee (38.8%). In their current positions at the time of survey response (Fig.3a), a portion of the CPFP alumni (84.9%) directly advised students, with most advising students less than half of the time (80.5%) and a small portion advising students for more than half (4.5%) of their time with variation based on job sector. Additionally, 96.6% of alumni served as mentors to others in their organizations (Fig.3b). Other mentoring activities reported in the past five years included 71.9% of alumni leading or contributing to multidisciplinary training programs [15] and 8.5% of alumni serving as editors of training manuals or textbooks.

Considering activities that are influenced by mentorship experiences, large portions of CFPF alumni performed research activities within and outside the field of cancer prevention and control (80.6% and 46.3%, respectively) that involved being a mentor and leading teams (Supplementary Table S1). Alumni also reported assuming a role as project leader (81.9%) and assumed leadership or management responsibilities (69.9%) as shown in Fig.3b. Within the open-ended reflection questions of the 2024 survey [15] (i.e., *“What do you consider to be the two or three most important accomplishments in your career?”* and *“What was the single most valuable aspect of the Cancer Prevention Fellowship Program to you?”*), alumni mentoring and networking accomplishments were highlighted frequently. For example, one of the CPFP alumni respondents wrote how being a mentor now is personally valued highly:

*“[An accomplishment highlighted as being] a Full Professor of Medicine with indefinite tenure and [the person] mentor[s] up and coming clinicians and senior trainees in both clinical Medicine and research…This was the primary reason why [the person] applied to CPFP in the first place.”*

This quote correlated with the observation that some of the CPFP alumni are incorporating mentorship practices that promote workforce development in the field across all job sectors. *“Mentoring dozens [of] fellows/jr staff”* and *“mentoring future generations of cancer researchers”* were also quotes among self-reported accomplishments of CPFP alumni. Themes of alumni accomplishments listed in Table 4 correlated well with time spent on various tasks within their current positions such as the tasks highlighted in Fig. 2 and Supplementary Table S1. As noted in Table 5, several CPFP alumni were recognized for mentoring and teaching activities in the past 5 years.

**Table 4:** Self-reported accomplishment of CPFP alumni^a^

Accomplishment Themes	Fellowship Alumni
Mentorship and Training (*n* = 30)	• Mentoring and training the next generation of researchers. (*n* = 23) • Advising students and junior scientists. (*n* = 5) • Developing programs for training and career development. (*n* = 2)
Collaboration and Networking (*n* = 28)	• Building national and international collaborative relationships. (*n* = 17) • Leading large coalitions and networks. (*n* = 2) • Organizing workshops and collaborative initiatives. (*n* = 9)

^a^Because this was an open response option, the responses were coded into overarching themes presented in the table above. Note that the themes from the optional responses may have come from more than one respondent, and/or one respondent having a combination of themes

**Table 5:** Awards and recognition for CPFP alumni in the past 5 years^a^

Award Type	Alumni (*n* = 93)
Special recognition, honorary awards, and alumni awards	22 (23.6%)
Teaching or Mentoring Awards	10 (10.8%)
NIH Director’s Awards and NIH Institute/Center Director’s Awards	22 (23.7%)
Other federal awards^b^	16 (17.2%)
Research and Scientific Achievements, conference awards, and fellowship selections	12 (12.9%)

^a^To reduce possibility of identifying respondents in small n values, the responses were coded into overarching award type categories presented in the table above

^b^Federal agencies included NIH (overall and institute/center/office types) and Food and Drug Administration (FDA)

## Discussion

The 2024 CPFP survey highlighted the value of mentorship throughout the training program and the importance of mentorship for alumni in their current public health positions within the field of cancer prevention and control. Since survey respondents sought out mentored research experience prior to gaining an entry-level field job, this study started with the assumption that the respondents also wanted to be mentored and have a career involving research. Furthermore, because the postdoctoral scholars have a slightly longer training period if participating in CPFP compared to applicants (Table 2), it was inferred that CPFP alumni experienced extended postdoctoral mentorship. This extended mentoring time in CPFP allowed for the opportunity to better establish these postdoctoral scholars as multidisciplinary cancer prevention and control experts, which was directly cited as a value of CPFP (Table 3). The impact of establishing their expertise was inferred by the types of honors and recognition alumni received (Table 5). Concurrently, CPFP alumni are effective mentors in their current jobs (Fig. 3 and Supplementary Table S1), while also contributing to the advancement of public health (Fig. 2).

When considering the career trajectory of CPFP alumni (Table 2 and Sherrer et al. [15]), the consideration of providing job sector specific mentorship arose to broaden career possibilities beyond research faculty in academia similar to past studies [16, 17]. For example, CPFP alumni in government leadership roles also supervise and mentor staff (Table 4; Fig. 3b, and Sherrer et al. [15]), which was directly quoted by a respondent in this study. Such roles, especially on multidisciplinary projects, can have products of mentoring and networking that include but are not limited to other special recognition and honorary awards, conference awards, and NIH awards stemming from alumni’s professional mentorship and/or team leadership (Table 5). Thus, a leadership role can involve mentorship activities beneficial to the next generation of researchers and leadership, especially in public health related fields. Notably, this training approach allows for an individuality component of mentorship desired by postdoctoral scholars [3, 18].

Examining training approaches also raises the question of mentorship being a primary or secondary goal of postdoctoral programs such as CPFP. Specifically, the popular view is that professional development is a strategic priority [3], but does that include mentorship training? If so, how consistent should this practice be across a field? To date, there are common elements of postdoctoral mentorship practiced and/or measured across various public health related fields. For example, depending on the type of training, postdoctoral scholars such as CPFP alumni (Table 3) may build a team of mentors to address multiple facets of their career advancement [8].

In addition, the quality of mentorship should be examined by the training program. Two-way transparency and honesty are suggested independent elements of mentorship, and these components must be in play during mentorship to allow for multidimensional support [3, 19]. Furthermore, heightened mentorship can occur when a mentor and a mentee have different experiences and/or when a postdoctoral scholar maintains dual roles as an informal mentor and a formal mentee [20]. These suggested practices increase the need to address the role of identity, personal agency, and well-being of postdoctoral scholars within the mentorship relationships [3, 9]. This last component is highlighted in the professional outcomes of CPFP alumni (Tables 4 and 5) as well as program aspects valued by alumni (Table 3).

Importantly, mentorship as measured in this study is intrinsically entwined into all aspects of CPFP, so evaluation of mentorship effectiveness can only be inferred at this point. If constructing relevant programmatic components, evaluation evidence collected has to be aligned within dimensions of mentorship to address postdoctoral scholars’ knowing-doing gap as mentors [3]. To achieve this objective, one should have mentorship success in mind at the onset of the mentor-mentee relationship and be aware that measuring its effectiveness will take time and over multiple iterations.

There are common elements collected when assessing postdoctoral mentorship. For example, the effectiveness of mentorship is frequently inferred by the overall career outcomes of mentees. When put into context of a specific employment sector such as academia, some studies identified predictors of successful career outcomes as being mostly publication records, evidence of balancing teaching-research tasks, and intended career at the beginning of the training program [12, 18, 21]. However, there is caution against using these metrics since they do not always directly align with postdoctoral scholar’s interest to pursue a specific type of career [12]. As highlighted here and in Sherrer et al. [15], directly measuring work retention in the field separate from professional recognition for high-quality mentorship performed by alumni is useful for describing mentorship impacts of a postdoctoral program. Other mentorship components could include low-risk high-reward opportunities and accountability for mentors as mentioned in Tables 4 and 5.

In terms of measuring mentees’ successes, one should value program participants’ shared experiences through transparent practices. There is evidence supporting postdoctoral scholars’ perceived success in intentional community-building programming [9, 22, 23] and direct research mentorship [8, 13]. Evidence has shown that mentorship addressing varying experiences and fostering professional independence strengthens mentees’ scientific identity, which is positively associated with career commitment and self-efficacy in career advancement [2, 12]. It is also worth noting that no CPFP alumni indicated that “perceived exclusion from the field” resulted in them no longer being in cancer prevention and control related work (Fig.1), which inferred strong scientific identity within the CPFP alumni community. 

### Limitations

Unfortunately, there are limitations to using the 2024 CPFP survey to examine mentorship within the program. As mentioned above, the quality of mentorship training and experiences for CPFP alumni and applicants as a function of career trajectory could not be determined at this time because the survey instrument was not designed to probe that type of information. The applicant response rate was lower than the alumni response rate, which also limited the extent to compare mentorship experiences with and without participation in CPFP. Additionally, it is noted that most CPFP alumni responses (99.5%) are counted as having completed at least one postdoctoral position (Table 2), and that not completing a single postdoctoral position (25.0% of applicants) may be due to a number of reasons. The most likely reasons could be that a postdoctoral position was not required to gain an entry-level position within the desired field, personal reasons taking priority, and/or professional interests had changed from their specialized field (Fig.1). The foremost reason is supported by respondents reporting that they did not spend any time in postdoctoral positions (Table 2). The same could be said for their professional interest changes being supported by having more than one discipline for work. Determining whether a postdoctoral position is necessary [17, 21] before starting an entry-level position such as tenure-track professorship or assistant scientist is a pivotal step for a mentee’s career success that should be open to discussion with a community of effective mentors.

## Conclusions

CPFP alumni reflected on their valued mentorship experiences and training during the program which, along with other factors inherent in the design of the fellowship program, aimed to facilitate workforce retention within the public health field of cancer prevention and control. Furthermore, the CPFP alumni regularly supported research participation by completing mentoring activities that aided the advancement of the next generation of scientists and contributed to workforce development. With iterative evaluations and aforementioned considerations, postdoctoral programs can implement and refine best practices for effective postdoctoral mentorship within the multidisciplinary field of cancer prevention and control.

## Supplementary Information

Supplementary Material 1.

## Data Availability

The aggregate data that support the findings of this study are available in the Methods, Results, and Supplementary Information sections of this article. Other data that are not shown in this article are available at reasonable request from the corresponding authors. The raw data are not publicly available due to privacy concerns and restrictions (ICF IRB exempt #2024-066).

## Abbreviations

CPFP: Cancer Prevention Fellowship Program
FDA: Food and Drug Administration
IRB: Institutional review board
MD: Doctor of Medicine
MPH: Master in Public Health
NCI: National Cancer Institute
NIH: National Institutes of Health
PhD: Doctor of Philosophy
STEMM: Science, technology, engineering, mathematics, and medicine

## References

[1] National Academies of Sciences, Engineering, and Medicine. A Population Health Workforce to Meet 21st Century Challenges and Opportunities: Proceedings of a Workshop. Applegate A, editor. Washington, DC: The National Academies Press. 2023. p. 104. 38354274

[2] Pfund C, Byars-Winston A, Branchaw J, Hurtado S, Eagan K. Defining Attributes and Metrics of Effective Research Mentoring Relationships. AIDS Behav. 2016;20(2):238–48. 27062425 10.1007/s10461-016-1384-z PMC4995122

[3] National Academies of Sciences, Engineering, and Medicine, Mentorship. Well-Being, and Professional Development in STEMM: Addressing the Knowing-Doing Gap: Proceedings of a Workshop—in Brief. Brown TK, Wynn ME, editors. Washington, DC: The National Academies Press; 2024. p. 13. 38452166

[4] Sun T, Drane D, McGee R, Campa H III, Goldberg BB, Hokanson SC. A national professional development program fills mentoring gaps for postdoctoral researchers. PLoS ONE. 2023;18(6):e0275767. 37315043 10.1371/journal.pone.0275767 PMC10266628

[5] Realmuto L, Daniel S, Jasani F, Weiss L, Bachrach C. Developing population health scientists: Findings from an evaluation of the Robert Wood Johnson Foundation Health & Society Scholars Program. SSM Popul Health. 2019;7:100373. 30809585 10.1016/j.ssmph.2019.100373 PMC6374691

[6] James AS, Gehlert S, Bowen DJ, Colditz GA. A Framework for Training Transdisciplinary Scholars in Cancer Prevention and Control. J Cancer Educ. 2015;30(4):664–9. 25510368 10.1007/s13187-014-0771-2 PMC4469633

[7] Krasna H, Kornfeld J, Cushman L, Ni S, Antoniou P, March D. The New Public Health Workforce: Employment Outcomes of Public Health Graduate Students. J Public Health Manag Pract. 2021;27(1):12–9. 30925525 10.1097/PHH.0000000000000976

[8] Subramanian S, Hutchins JA, Lundsteen N. Bridging the gap: increasing collaboration between research mentors and career development educators for PhD and postdoctoral training success. Mol Biol Cell. 2022;33(2):pe1. 35041468 10.1091/mbc.E21-07-0350 PMC9236141

[9] Lee H, Salcedo J, Chen K, Anderson AJ. This is Why We All Show Up: How Supporting Youth Cultivates Hope, Purpose, and Well-Being of Adult Mentors. J Community Psychol. 2025;53(2):e23182. 39895000 10.1002/jcop.23182 PMC11788525

[10] National Postdoctoral Association. About the National Postdoctoral Association. https://www.nationalpostdoc.org/page/About. Accessed 30 Nov 2025.

[11] Polka JK, Krukenberg KA, McDowell GS. A call for transparency in tracking student and postdoc career outcomes. Mol Biol Cell. 2015;26(8):1413–5. 25870234 10.1091/mbc.E14-10-1432 PMC4395122

[12] Afonja S, Salmon DG, Quailey SI, Lambert WM. Postdocs’ advice on pursuing a research career in academia: A qualitative analysis of free-text survey responses. PLoS ONE. 2021;16(5):e0250662. 33956818 10.1371/journal.pone.0250662 PMC8101926

[13] Smyth SS, Coller BS, Jackson RD, Kern PA, McIntosh S, Meagher EA, et al. KL2 scholars’ perceptions of factors contributing to sustained translational science career success. J Clin Transl Sci. 2022;6(1):e34. 35433037 10.1017/cts.2021.886 PMC9003634

[14] Faupel-Badger JM, Nelson DE, Izmirlian G, Ross KH, Raue K, Tsakraklides S, et al. Independent Association of Postdoctoral Training with Subsequent Careers in Cancer Prevention. PLoS ONE. 2015;10(12):e0144880. 26659381 10.1371/journal.pone.0144880 PMC4682206

[15] Sherrer SM, Faupel-Badger JM, Zanetti KA, Bowles HR, Dent K, Swigart T, ZuWallack R, Castle PE. Outcomes of the NCI cancer prevention fellowship program in training scholarly multidiscplinary public health professional leaders. Sci Rep. 2026. 10.1038/s41598-026-45502-4. 10.1038/s41598-026-45502-4 PMC13219630 41946903

[16] Mitic R, Okahana H. Closing Gaps in our Knowledge of PhD Career Pathways: How Do Biological and Life Sciences PhD Holders Transition into the Workforce. CGS Res Brief. 2020:1–6. Available from: https://cgsnet.org/wp-content/uploads/2022/02/CGS_ResearchBrief_BioMed_v5.pdf.

[17] National Research Council (US). Committee to Study the National Needs for Biomedical, Behavioral, and Clinical Research Personnel. 3, Basic Biomedical Sciences. Research Training in the Biomedical, Behavioral, and Clinical Research Sciences. Washington, DC: National Academies; 2011. pp. 27–50.

[18] Rybarczyk BJ, Lerea L, Whittington D, Dykstra L. Analysis of Postdoctoral Training Outcomes That Broaden Participation in Science Careers. CBE Life Sci Educ. 2016;15(3):ar33. 10.1187/cbe.16-01-0032 PMC5008880 27543634

[19] Asquith P, McDaniels M, Baez A, Corsino L, Fillingim R, Rubio D, et al. Advancing the Science of Mentorship: Future Directions for Sustainable Implementation and Evaluation of Mentorship Education for the Clinical and Translational Science Workforce. J Clin Transl Sci. 2024;8(1):e54. 38577552 10.1017/cts.2024.497 PMC10993062

[20] Higino G, Barros C, Bledsoe E, Roche DG, Binning SA, Poisot T. Postdoctoral scientists are mentors, and it is time to recognize their work. PLoS Biol. 2023;21(11):e3002349. 37917597 10.1371/journal.pbio.3002349 PMC10621833

[21] Bessudnov A, Guardiancich I, Marimon R. A statistical evaluation of the effects of a structured postdoctoral programme. Stud High Educ. 2015;40(9):1588–604.

[22] Eisen A, Eaton DC. A Model for Postdoctoral Education That Promotes Minority and Majority Success in the Biomedical Sciences. CBE Life Sci Educ. 2017;16(4):ar65. 10.1187/cbe.17-03-0051 PMC5749967 29196426

[23] Goldberg BB, Bruff DO, Greenler RM, Barnicle K, Green NH, Campbell LE, et al. Preparing future STEM faculty through flexible teaching professional development. PLoS ONE. 2023;18(10):e0276349. 37824586 10.1371/journal.pone.0276349 PMC10569627

