# Supplementary material for: Outcomes of the NCI cancer prevention fellowship program: a focus on mentorship within a multidisciplinary public health field
Source: BMC Med Educ. 2026 May 13;26:1112. doi: 10.1186/s12909-026-09387-1 (PMC13352972; doi:10.1186/s12909-026-09387-1)
Supplement: Supplementary file 1 — Supplementary Material 1. [file 12909_2026_9387_MOESM1_ESM.pdf]

Supplementary Information:

Outcomes of the NCI cancer prevention fellowship program:

a focus on mentorship within a multidisciplinary public health field

Shanen M. Sherrer[1,2]*, Jessica M. Faupel-Badger[3], Krista A. Zanetti[4], Heather R. Bowles[1], Philip E. Castle[1]*

Affiliations noted:

[1]Cancer Prevention Fellowship Program, National Cancer Institute, 9606 Medical Center Drive, Rockville, MD, USA.

[2]AAAS Science & Technology Policy Fellow, Department of Chemistry and Biochemistry, St. Mary's College of Maryland, St. Mary's City, MD, USA.

[3]Division of Research Capacity Building, National Institute of General Medical Sciences, Bethesda, MD, USA.

[4]Office of the Director, National Institutes of Health, Bethesda, MD, USA.

*Correspondence:
Shanen Sherrer smsherrer@smcm.edu
Philip Castle philip.castle@nih.gov

Fig. S1.

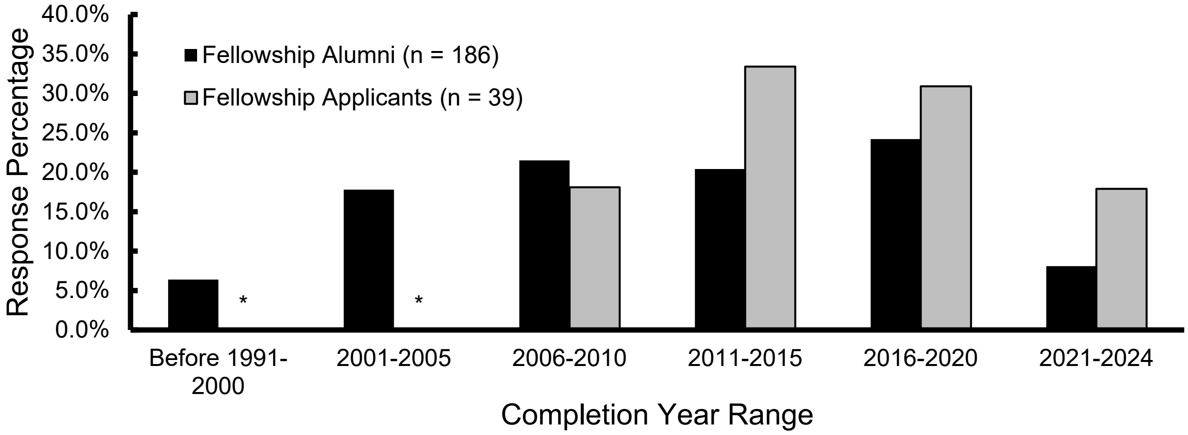

Fig. S1. Completion year of the most recent postdoctoral fellowship overlap between CPFP alumni (black bars, *n* = 186) and applicants (grey bars, *n* = 39). The peak years of postdoctoral training completion for CPFP alumni are between 2001 and 2020 while the peak years of postdoctoral training completion for CPFP applicants (grey bars) are between 2006 and 2024. The * mark denotes data suppression due to small response amounts (*n* < 10). Data was derived from Sherrer et al [15].

Fig. S2.

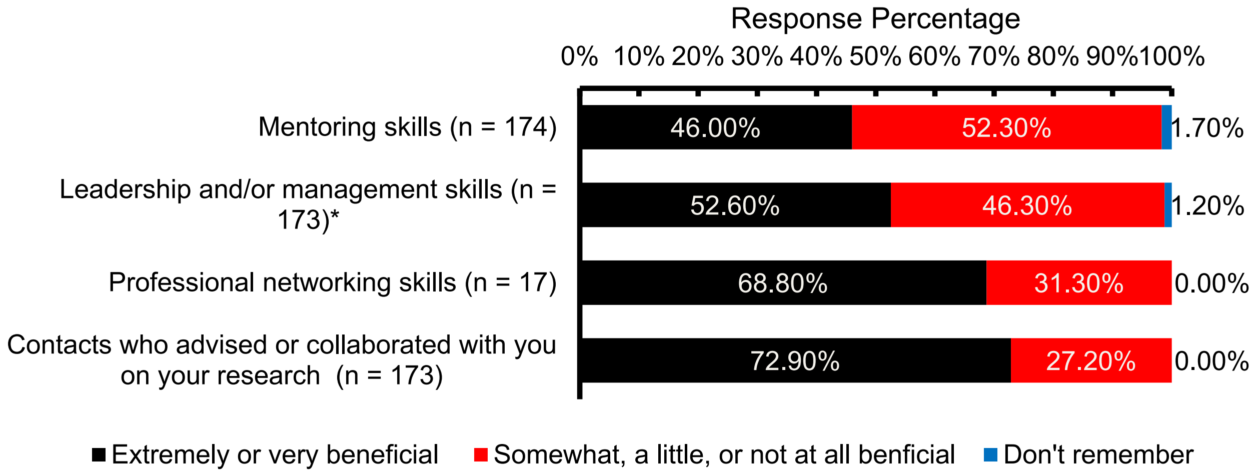

Supplementary Fig. S2. Beneficial aspects of CPFP according to alumni's career. CPFP alumni indicated specific aspects of the program (vertical items) related to either mentoring and/or networking that were somewhat, a little, or not at all beneficial (red bars); extremely or very beneficial (black bars); or don't remember (blue bars). The * mark denotes data from Sherrer et al [15].

Table S1.

Supplementary Table S1. Percentage of time spent on research activities for CPFP alumni.	
**Item**	**CPFP alumni who spend at least 50% of their time on item (%)[a]**
Research and research support activities in general	145 (80.6 % out of 180)
Research and research support activities in cancer prevention and control[b]	82.0 (46.3 % out of 177)

[a]Activities included conducting research directly as well as research management, monitoring, reviewing, funding, analysis, dissemination, mentoring, and other research support activities.

[b]Data derived from Sherrer et al [15].
